# Intranasal delivery of engineered anti-SARS-CoV-2 extracellular vesicles therapeutically represses lung infection and inflammation

**DOI:** 10.1007/s13346-025-01922-9

**Published:** 2025-07-17

**Authors:** Adi Idris, Surya Shrivastava, Wenqing Gao, Aroon Supramaniam, Yaman Tayyar, Nicholas P. West, Gabrielle Kelly, Dhruba Acharya, Nigel A.J. McMillan, Kevin V. Morris

**Affiliations:** 1 Institute of Biomedicine and Glycomics and School and Pharmacy and Medical Sciences, Griffith University, Southport, QLD Australia; 2 Centre for Immunology and Infection Control, School of Biomedical Sciences, Queensland University of Technology, Brisbane, QLD Australia; 3 Center for Gene Therapy, City of Hope, Beckman Research Institute and Hematological Malignancy and Stem Cell Transplantation Institute, Duarte, CA USA; 4 Centre for Genomics and Personalised Health, School of Biomedical Sciences, Queensland University of Technology, Kelvin Grove, Brisbane, QLD Australia

**Keywords:** SARS-CoV-2, Spike protein, Neural stem cell, VHH72, Extracellular vesicles

## Abstract

Extracellular vesicles (EVs) are amenable to genetic engineering in that EVs can be endowed with surface armaments that can directly bind to target molecules or receptors. We previously developed HEK293 cell-derived EVs that contain a novel fusion tetraspanin protein, CD63, embedded within a highly conserved anti-SARS-CoV-2 nanobody, VHH72. These anti-SARS-CoV-2-enriched EVs bind SARS-CoV-2 spike protein and can functionally neutralize SARS-CoV-2 in vitro. Here, we extend our observations in vivo using EVs derived from neural stem cells (NSCs) and demonstrated the antiviral effectiveness of these direct-acting EVs in the lungs of SARS-CoV-2 infected mice when administered intranasally post-infection. Using NanoString-based immune transcriptomics we showed that these EVs exert mild anti-inflammatory effects on SARS-CoV-2 infected lungs. This is the first demonstration of the effective use of intranasally delivered EVs ladened with anti-SARS-CoV-2 nanobodies in vivo.

**Graphical Abstract:**

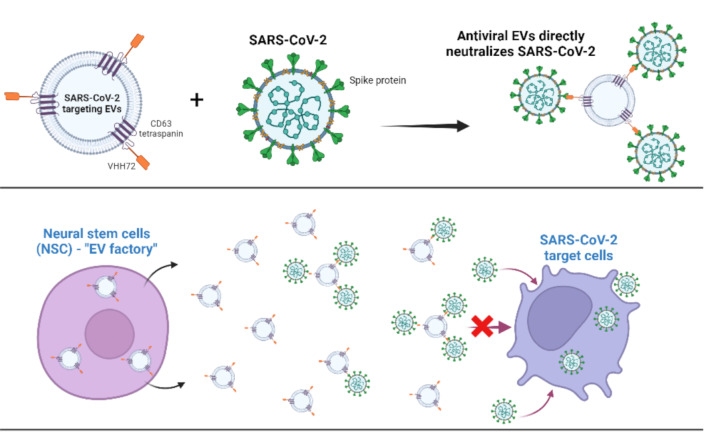

**Supplementary Information:**

The online version contains supplementary material available at 10.1007/s13346-025-01922-9.

## Introduction

Currently approved antivirals against SARS-CoV-2 are designed to either extracellularly neutralize the virus (monoclonal antibody approach e.g., sotrovimab) or stop viral replication intracellularly (nucleoside analogues e.g., molnupiravir). Though antivirals alone are effective in improving clinical outcomes for COVID-19 patients, combination of an anti-inflammatory drug with an antiviral drug can reduce recovery time and accelerate clinical improvement in hospitalized COVID-19 patients [1]. Indeed, combination therapy of a protease inhibitor antiviral, ensitrelvir, and corticosteroids was shown to reverse the delayed antiviral monotherapy clinical response in SARS-CoV-2 infected hamsters [2], demonstrating the clinical value of combination therapy with antiviral and anti-inflammatory drugs for the treatment of COVID-19. We previously developed highly innovative SARS-CoV-2 therapeutic approaches using extracellular vesicles (EVs) [3–5] and against other non-respiratory viruses [6]. Our EV platform consists of a means to target SARS-CoV-2 directly using a modified CD63, a ubiquitous EV transmembrane protein, fused to receptor targeted nanobodies. We have previously embedded the camelid-derived nanobody, VHH72, a nanobody directed to SARS-CoV-2 spike protein [7] from multiple variants [8, 9], onto CD63 of HEK293 cell-derived EVs (HEK VHH72-EVs) [5]. We showed that HEK VHH72-EVs can directly neutralize multiple live SARS-CoV-2 variants in vitro. However, we have yet to functionally test these EVs in vivo. We have also used EVs derived from neural stem cells (NSCs) loaded with antiviral long antisense RNA (asRNA) and showed that it can repress SARS-CoV-2 replication in the lungs of mice and dampen inflammation in vitro [4]. Armed with this knowledge and our capability in engineering VHH72 on the surface of EVs [5], we speculated whether we could develop an antiviral therapy using anti-inflammatory EVs that can directly neutralize viruses in vivo. Here, we engineered anti-inflammatory NSC EVs to be endowed with a SARS-CoV-2 spike protein targeting nanobody (VHH72) and demonstrated that intranasal administration of these EVs reduced lung infection and SARS-CoV-2 driven tissue inflammation.

## Methodology

### Plasmids

EXOtic device plasmids, pDB68 (Connexin43 S368A), pDB30 (CD63-nLuc), were kind gifts from Dr Martin Fussenegger [10], and VHH72 (pcDNA-CD63-VHH [5]).

### Cell lines

Vero E6 and neural stem cells (NSC) (A4 lineage) and HEK293 cells were cultured in Dulbecco’s modified Eagle’s medium (DMEM) (Thermo Fisher Scientific) supplemented with 10% fetal bovine serum (FBS) and incubated at 37 °C and 5% CO^2^.

### Virus

SARS-CoV-2 Wuhan (Ancestral - VIC1) strain was obtained from the Peter Doherty Institute for Infection and Immunity and Melbourne Health, Victoria, Australia and cultured in Vero E6 cells. All SARS-CoV-2 infection work was conducted in a BSL3 approved animal facility at Griffith University.

### Viral plaque and immunoplaque assays

For viral plaque assays, Vero E6 cells were infected with SARS-CoV-2 for 1 h before overlaying with 1% methylcellulose (MC) agar- viscosity (4,000 centipoises) (Sigma- Aldrich). Cells were incubated for 4 days at 37 °C before fixing in 8% formaldehyde and stained with 1% crystal violet to visualize plaques. Viral immunoplaque assays for SARS-CoV-2 were performed on Vero E6 cells as described previously [11] using recombinant monoclonal antibodies that recognizes the same SARS-CoV-2 spike protein (CR3022) epitope that VHH72 recognises [8, 9].

### Spike protein binding assay

This assay was done as described previously [5]. HEK293 cells were transfected with either a control vector or a SARS-CoV-2 spike-expressing vector (Sino Biological, Beijing, China). After 48 h, equal numbers of VHH72-CD63 or control CD63 EVs were added to the cells and incubated at 37 °C and 5% CO_2_ for 4 h and washed with PBS. Luciferase activity was determined using the Nano-glo luciferase assay system (Promega).

### In vitro SARS-CoV-2 neutralization assay

EVs or the monoclonal anti-SARS-CoV-2 RBD antibodies (clone nos. CB6 and 5309) were incubated with 250 PFUs of SARS-CoV-2 at the described concentrations for 30 min at RT before infecting Vero E6 cells for 1 h at 37^o^C. The virus was then removed, and the wells were layered with MC agar. The numbers of plaques were assessed 4 days after infection.

### Viral copy number determination

To determine viral copy numbers in infected tissues, digital PCR against the N gene of SARS-CoV-2 (CDC primers from IDT - SARS-CoV-2 N1) was performed in Quant-Studio 3D Digital PCR 20 K chips (Thermo Scientific) on a ProFlex 2×Flat Block Thermal Cycler (Thermo Scientific). Results are analyzed on the QuantStudio 3D Analysis Suite software (Thermo Scientific) and expressed as viral copies per µl of template RNA.

### EV production and characterization

Control and anti-SARS-CoV-2 targeting EVs in this study were produced using the following plasmids from the EXOtic packaging system as we have done previously in HEK293 cells [5]:

**Table:** 

DB30 (Control EVs)	pDB30 (CD63-nLuc) + pDB68 (connexion EV release)
VHH (Anti-CoV-2 EVs)	VHH72 (pcDNA-CD63-VHH72) + pDB68 (connexion EV release)

Producer cells were transfected using Lipofectamine 3000 (Thermo Fisher Scientific) according to manufacturer instructions over 24 h before washing cells with DMEM and replacing media with DMEM + 10% EV-depleted FBS (Thermo Fisher Scientific). Supernatant was collected at 48–96 h later and centrifuged at 300 x g for 10 min at 4^o^C. The viability of the cells was determined at the time of EV collection, which was greater than 90%. Supernatants were transferred to a new 50-mL conical tube and further centrifuged at 2000 x g for 20 min at 4^o^C before passing the supernatant through a 0.45-mm filter (Millipore). The filtered supernatant was then ultracentrifuged at 100,000 x g for 120 min at 4^o^C on a SW32Ti rotor in a Beckman XL-900 ultracentrifuge to pellet the EVs and were resuspended in PBS. The final precipitate was resuspended in sterile PBS and passed through 0.22-micron Ultrafree^®^ Centrifugal Filter Units (Millipore Sigma) before storing at − 20 °C until used. A recent study has shown that EVs stored at 4 °C or -20 °C for short periods do not significantly change compared to those stored at -80 °C [12]. EVs were then quantified using Nanoparticle Tracking Analysis (NTA) on a NanoSight (Malvern Panalytical) (1/4000 dilution) (Figure S1). A 488-nm laser was used to detect the EVs (slide shutter level = 1,259, slider gain = 366, syringe pump speed = 30) using a flow-cell top plate module. To determine particle count, a threshold setting = 3 was used. We then further confirmed that these are CD63-positive EVs by determining luciferase activity via the Nluc-tagged to CD63 relative to ultracentrifuged supernatant from untransfected cells (i.e., no EVs) as done previously [4]. On average we routinely collect ~ 10–12 × 10^9^ EVs per 10 cm plate of NSCs (1–2 × 10^6^ cells) over a 96 h period.The particle size, polydispersity index (PDI) and zeta potential index of the EVs were obtained using Zetasizer Nano ZS (Malvern Instruments, Malvern, UK) following appropriate dilution in PBS (Figure S1) to overcome the reported limitations associated with measuring EV physio-characteristics using the NTA [13, 14]. The negative zeta potential of the EVs (surface charge) confirms the colloidal stability of EVs and values consistent with what was previously reported [15]. All measurements were carried out at room temperature.

### Transmission electron microscopy

NSC EVs were visualized using transmission electron microscopy with negative staining. Prior to imaging, NTA determined EV concentrations at 2.07 × 10^9^ particles/mL. For TEM, samples were diluted 1:2 and 1:10 in MilliQ water, yielding working concentrations of 1.04 × 10⁹ and 2.07 × 10⁸ particles/mL, respectively. Copper grids (200–300 mesh, formvar/carbon-coated) were glow-discharged for 30 s at 400 mTorr (Evactron) to enhance hydrophilicity. A 5 µL droplet of EV suspension was placed on parafilm, and the grid (dark side down) was floated on the droplet for 3 min. Excess was wicked away using filter paper, and grids were immediately stained with 2% aqueous uranyl acetate for 2 min. Grids were then air-dried. Imaging was performed using a JEOL 1400 TEM (Jeol Pty Ltd, Japan), operated at 120 kV mounted with a 2 K TVIPS CCD camera at the Central Analytical Research Facility (CARF), Queensland University of Technology (QUT).

### EV biodistribution in mice

NSC EVs were diluted in sterile PBS were complexed with DiIC18(7);1,1′-dioctadecyl-3,3,3′,3′-tetramethylindotricarbocyanine iodide (DIR), a lipophilic, near-infrared fluorescent cyanine dye at a final concentration of 1.55ng/µl DIR. A total of 10^8^ NSC EVs were administered to C57BL/6 mice intranasally (20µL). Fluorescence was detected using a single photon animal imager at various timepoints post-administration.

### SARS-CoV-2 in vivo work

K18-hACE2 mice (3–4 months old) were purchased from the Jackson Laboratory (Bar Harbor, ME) and bred in-house at the Griffith University Animal Resource Center. Mice were intranasally infected with 10^4^ plaque forming unit (PFU) (20µL total volume) of live SARS-CoV-2 while under isoflurane anesthesia. Mice were subsequently treated with EVs either retro-orbitally (intravenous) (100µL total volume) or intranasally (20µL total volume) while under isoflurane anesthesia. Mice were weighed and scored daily until the experimental endpoint for disease progression. The well-being of mice was evaluated based on locomotion, behaviour, and appearance.

### NanoString gene expression analysis

Immune gene expression analysis was undertaken using the NanoString nCounter analysis system (NanoString Technologies, Seattle, WA) and the commercially available nCounter Mouse Inflammation Panel. The Mouse Inflammation panel contains 248 genes of key inflammatory pathways and 6 reference/housekeeping genes. The nCounter system directly detects and counts single-stranded nucleic acid via reporter probes affixed with fluorophore barcodes and biotinylated capture-probes attached to microscopic beads. Probes are then affixed to lanes in cartridges and read in a digital scanner. Following the manufacturer’s protocol, 100 ng of total RNA extracted from tissue was hybridised with probes at 65 °C for 20 h before being inserted into NanoString Prep Station where the target-probe complex was immobilised onto the analysis cartridge. Cartridges were read by the nCounter Digital Analyser for digital counting of molecular barcodes corresponding to each target at 555 fields of view.

### Nanostring data analysis

Gene expression data was analysed using a combination of the Advanced Analysis Module in the nSolver™ Analysis Software version 4.0 from NanoString Technologies (NanoString Technologies, WA, USA) or the Limma package in the R Statistical Computing Environment. nSolver enables quality control (QC), normalisation, differential gene expression (DGE), Pathview Plots and immune cell profiling. Negative and positive controls included in probe sets were used for background thresholding, and normalizing samples for differences in hybridization or sample input respectively. Data was corrected for input volume via internal housekeeping genes using the geNorm algorithm. Genes that were expressed below 20 counts in more than 90% of samples were excluded from analysis. Differential gene expression between the treatment groups was determined using a variance stabilized t-test. Pathway analysis was undertaken using the Gene Ontology and Kyoto Encyclopedia of Genes and Genomes (KEGG).

### Quantitative real-time PCR (qRT-PCR)

Total RNA was extracted from mouse lung tissues for gene expression analysis. The relative mRNA levels of key inflammatory genes were performed using the UniPeak U One Step RT-qPCR SYBR Green Kit (Vazyme) and Rotor-Gene Q (Qiagen) platform. The specific primer pairs used are listed in Table 1. Gene expression was normalized to the endogenous control β-actin. All reactions were performed in triplicate, and relative gene expression was calculated using the 2 ^ (-ΔΔCt) method.

**Table 1:** qRT-PCR primers used in this study

Gene	Forward primer	Reverse primer
IFN-α	TGCCCAGCAGATCAAGAAGG	TCAGGGGAAATTCCTGCACC
IFN-β	GTACAACAGCTACGCCTGGA	GAGTCCGCCTCTGATGCTTA
IFN-γ	TCAGGCCATCAGCAACAACA	GTGGACCACTCGGATGAGC
TNF-α	GGTGCCTATGTCTCAGCCTCTT	GCCATAGAACTGATGAGAGGGAG
IL-6	TACCACTTCACAAGTCGGAGGC	CTGCAAGTGCATCATCGTTGTTC
MCP-1	GCTACAAGAGGATCACCAGCAG	GTCTGGACCCATTCCTTCTTGG
β-actin	GCTGTGCTATGTTGCTCTAG	CGCTCGTTGCCAATAGTG

### Statistical analysis

All statistical analyses were performed using the statistical software package GraphPad Prism v9 and described in detail in respective figure legends.

## Results and discussion

**Fig. 1:**
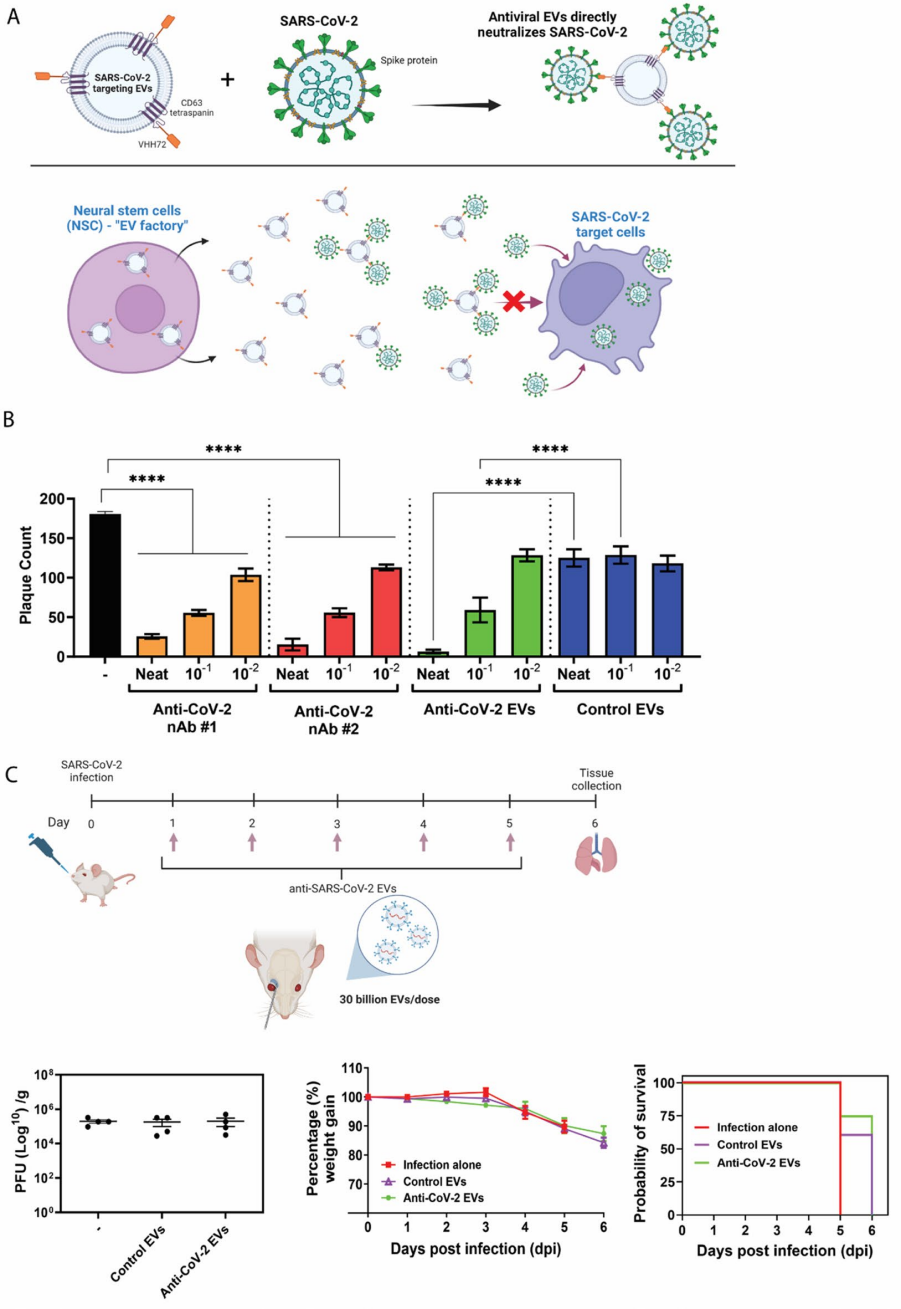
EVs imbued with VHH72 nanobody can directly neutralise SARS-CoV-2 infection in vitro but fails to reduce lung infection when administered intravenously in vivo. (**A**) Schematic of therapeutic approach of using NSC-derived EVs expressing VHH72 to directly neutralize SARS-CoV-2 infectious viral particles. (**B**) EVs or neutralizing antibodies were used undiluted or serially diluted 1-fold before mixing with 250 PFU of live SARS-CoV-2 for 30 min at RT before infecting Vero E6 cells for 1 h at 37^o^C. Virus and neutralizing antibody/EV mixtures were then removed before performing a viral plaque assay for 5 days. Neat EV concentration = 2.5 × 10^7^ exosomes/µL. Two clones of monoclonal neutralizing antibodies against SARS-CoV-2 (clone #1–5309 and clone #2– CB6) were used as a positive control (Neat Ab concentration = 0.01 mg/ml). Triplicate treated cells are shown with the standard error of the mean (SEM) of triplicate treatments. *p* < 0.0001 (****), One-way ANOVA test. (**C**) 7–13-week-old K18-hACE2 mice were intranasally infected with 10^4^ PFU of SARS-CoV-2. Mice were intravenously administered with 30 billion NSC EVs in 100 µL of PBS at the indicated days post-infection (dpi). At 6 dpi mice were euthanized, lung tissues were harvested and homogenized for viral immunoplaque assays. The amount of infectious virus particles in lung tissues at 6dpi were determined by viral immunoplaque assays on Vero E6 cells and expressed as PFU per gram (g) of tissue. represents the average ± SEM of 4 mice. Mice were weighed and scored daily until the experimental endpoint for disease progression. Body weight (weight change) and probability of survival were evaluated at the indicated dpi. Mice that lost > 20% of their initial body weight were humanely euthanized and plotted as non-survivors. Each data point represents the average ± SEM of 4 mice

**Fig. 2:**
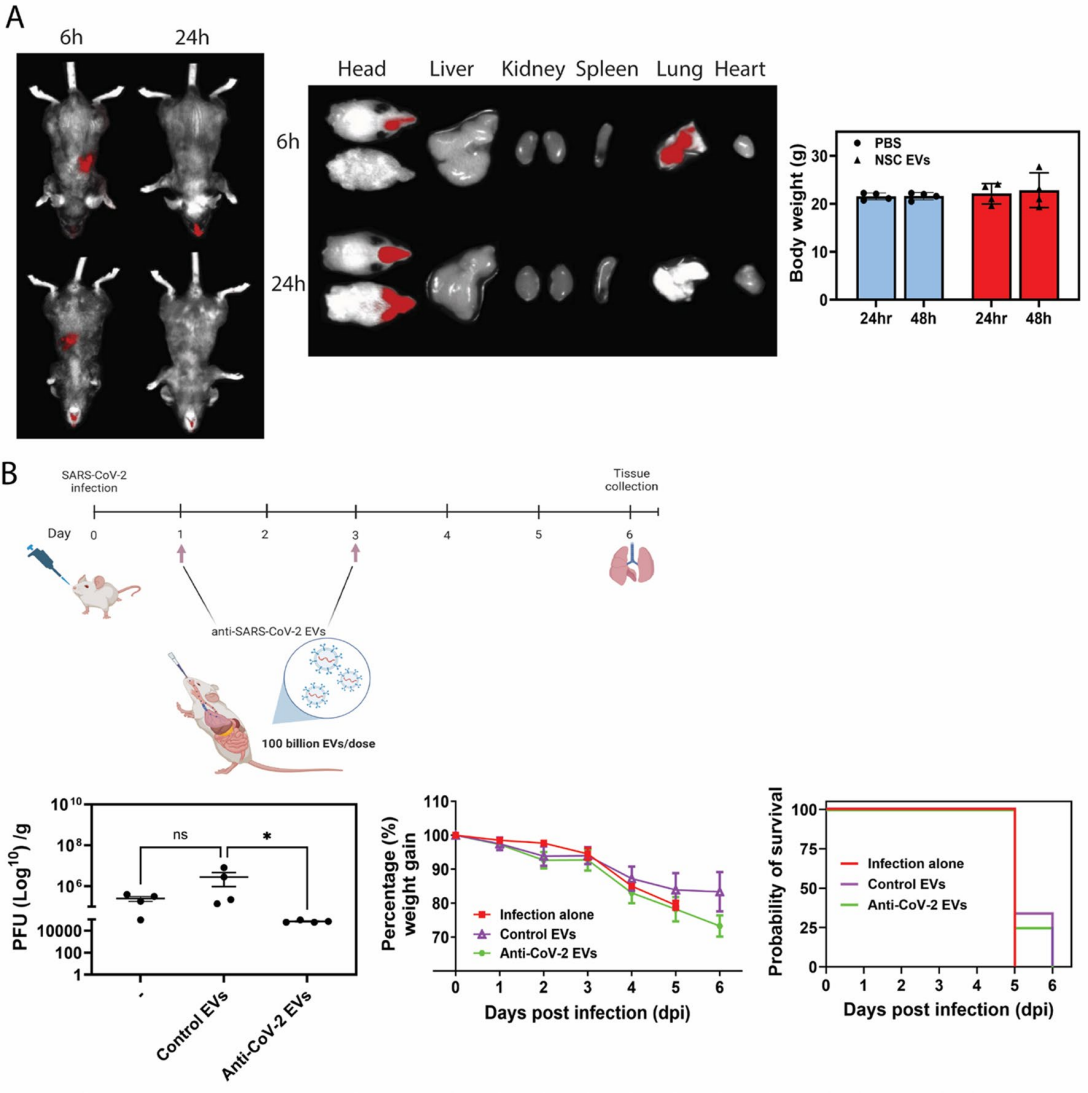
Intranasal delivery of SARS-CoV targeting EVs localizes to the lungs and significantly reduced SARS-CoV-2 lung infection. (**A**) 10^8^ NSC EVs complexed with DIR were administered to C57BL/6 mice by intranasal administration (20µL). Fluorescence was detected using a single photon animal imager at the indicated timepoints post-administration. Raw body weigh data is shown. Each data point represents the average ± SEM of 4. (**B**) 7–13-week-old K18-hACE2 mice were intranasally infected with 10^4^ PFU of SARS-CoV-2. Mice were administered with 100 billion NSC EVs in 20 µL of PBS by intranasal administration at 1 and 3dpi. At 6 dpi mice were euthanized, lung tissues were harvested and homogenized for viral immunoplaque assays. The amount of infectious virus particles in lung tissues at 6dpi were determined by viral immunoplaque assays on Vero E6 cells and expressed as PFU per gram (g) of tissue. Mice were weighed and scored daily until the experimental endpoint for disease progression. Mice that lost > 20% of their initial body weight were humanely euthanized and plotted as non-survivors. Each data point represents the average ± SEM of 4 mice. Each data point represents the average ± SEM of 4 mice. *p < 0.05, One-way ANOVA test

We previously showed that HEK VHH72-EVs can neutralize multiple emerging SARS-CoV-2 variants of concern [5]. Using the same bioengineering approach, we developed VHH72 decorated EVs (anti-CoV-2 EVs) derived from NSCs which preferentially binds SARS-CoV-2 spike protein (Figure S2). To test the ability of NSC-derived anti-CoV-2 EVs to neutralize live SARS-CoV-2 in vitro (Fig. 1A), we incubated SARS-CoV-2 with anti-CoV-2 EVs before exposing to a monolayer of VeroE6 cells. Compared to non-VHH72 EVs (control EVs), anti-CoV-2 EVs significantly reduced SARS-CoV-2 infection to the same degree as anti-SARS-CoV-2 neutralizing antibody controls suggesting that these EVs can directly neutralize infectious SARS-CoV-2 viral particles (Fig. 1B). Several monoclonal antibodies (mAbs) targeting SARS-CoV-2 spike protein have been developed since the pandemic. However, the clinical use of it has weaned over time due to the rapidly mutating spike protein as new variants emerge over time [16]. Over time, SARS-CoV-2 variants have acquired mutations in the immunodominant receptor binding motif (RBM) [17]. VHH72 targets highly conserved and non-variable spike protein receptor binding domain (RBD) epitopes and is cross-reactive across several currently circulating SARS-CoV-2 variants [8, 9]. Indeed, the in vivo potential of prophylactically administered VHH72-human immunoglobulin G1 Fc domain fusion in SARS-CoV-2 infection models via both the intraperitoneal and intranasal routes can clinically rescue the animals and modestly reduce infectious lung viral loads [18]. The prophylactic treatment strategy can be rationalized to neutralize infectious viral particles before it can enter host cells. However, this approach may not be clinically beneficial if targeting SARS-CoV-2 infection post-exposure. On the other hand, a therapeutic approach using direct binding neutralizing agents could inhibit the viral egress [19]. Here, we wanted to test the effect of our anti-CoV-2 EVs in SARS-CoV-2 infected mice when administered post-infection (therapeutically). Mice were infected with SARS-CoV-2 overnight before administering five daily doses of anti-CoV-2 EVs by intravenous infusion. Anti-CoV-2 EV treatment failed to reduce lung viremia in the lungs of infected mice (Fig. 1C). We reasoned that this could be due to the poor NSC EV distribution in mouse lungs when delivered intravenously [4]. Hence, we resorted to testing the therapeutic effect of these anti-CoV-2 EVs by delivering via the intranasal route. Intranasal delivery of NSC EVs resulted in favourable lung biodistribution and did not negatively impact the mice even after 48 h post-EV intranasal administration (Fig. 2A). Consistent with this, we observed a significant decrease in lung viral load by almost two log fold in mice intranasally treated with only two doses of anti-CoV-2 EVs (Fig. 2B, Figure S3), underlining the suitable utility of intranasal delivery to achieve an ideal therapeutic outcome in the lower respiratory tract. Despite this, we are aware that the antiviral effect observed is not close to maximal. We argue this may have to do with our engineered EVs having a large diameter (Figure S1), making it difficult for the EVs to pass beyond lung bronchioles to reach the alveoli. This could also account for the negligible antiviral effect with intravenously infused EVs (Fig. 1C). It is entirely possible that our EVs are unable to efficiently penetrate the alveolar epithelium following systemic intravenous injection. Indeed, our previous biodistribution work with NSC EVs supports this argument [4].

Though clinical improvement in animals (via body weight) was not observed in intranasally treated anti-CoV-2 groups (Fig. 2B), we noticed mild survival advantage in infected mice administered either intranasally or intravenously with NSC EVs (both anti-CoV-2 and control EVs) when compared to infected mice alone (Figs. 1B and 2B). It is important to note that the degree of survival advantage cannot be directly compared between the groups that received NSC EVs via different routes of administration as the dose (intravenous– 30 billion EVs/dose vs. intranasal– 100 billion EVs/dose) and administration frequency (intravenous– total of five daily doses vs. intranasal– total of two doses every two days) varies greatly (Figs. 1B and 2B). Nonetheless, we suspect that mild survival advantage (albeit not statistically significant by log-rank test) in infected mice administered both anti-CoV-2 and control EVs could be due to the anti-inflammatory properties of these NSC-derived EVs [4].Therefore, we explored the potential for NSC EVs to dampen lung inflammation.

**Fig. 3:**
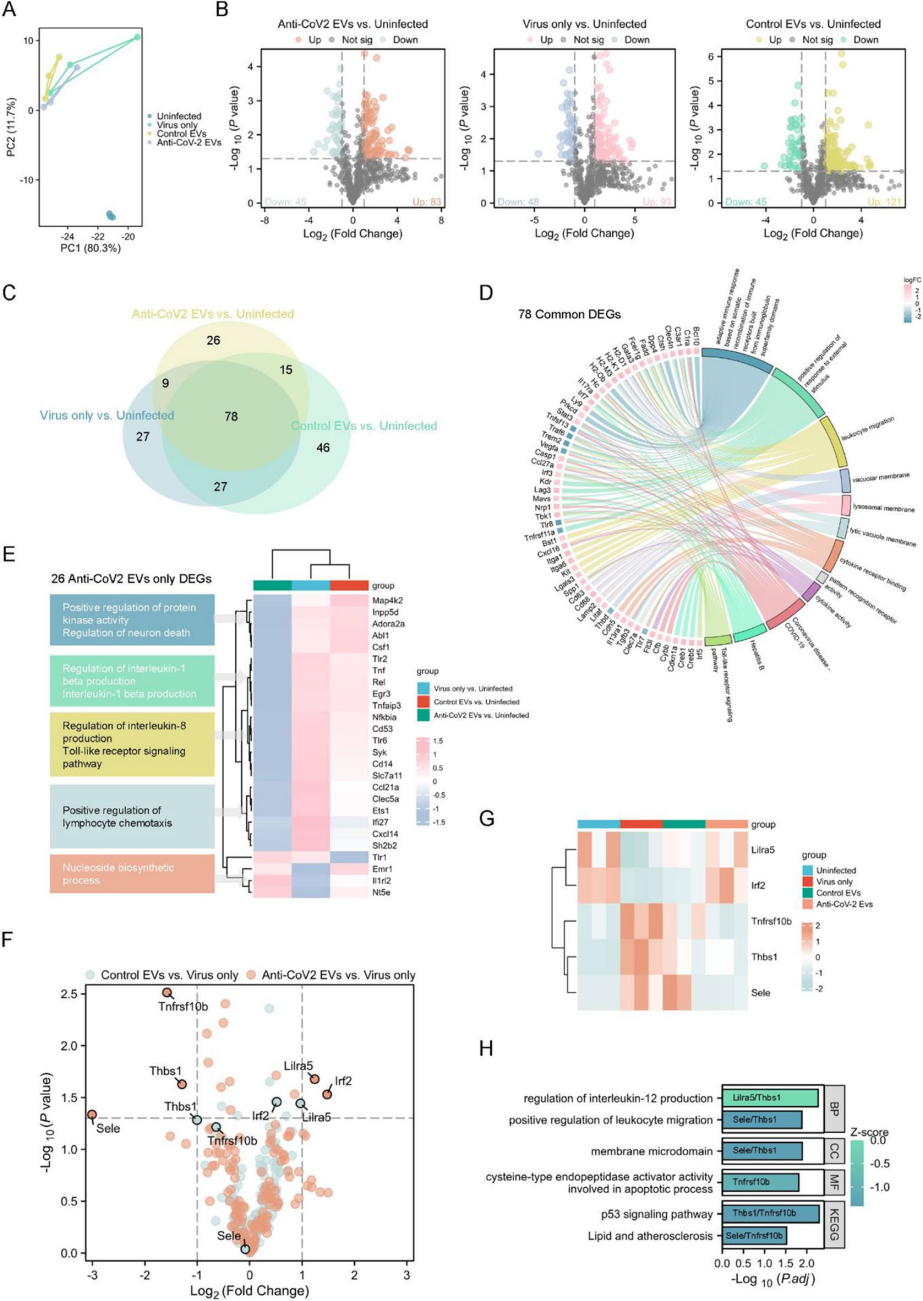
Intranasal administration of targeting EVs in SARS-CoV-2 infected mice alters the lung inflammatory transcriptomic profile. (**A**) Principal Component Analysis (PCA) of the transcriptomic profiles of EVs isolated from four experimental groups: uninfected, virus only, control EVs, and anti-CoV-2 EVs. Data is representative of *n* = 3 mice per experimental group. (**B**) Volcano plots show differential gene expression analysis between different experimental groups. Significant genes are represented in different colors, while non-significant genes are shown in grey. The horizontal dashed line represents a p-value cutoff of 0.05, and the vertical dashed lines represent a fold change cutoff of ± 1. Data is representative of *n* = 3 mice per experimental group. (**C**) Venn diagram illustrates the overlap of differentially expressed genes (DEGs) between different experimental groups. Data is representative of *n* = 3 mice per experimental group. (**D**) Circos plot of the 78 common DEGs, annotated with their involvement in cellular compartments and biological functions. Data is representative of *n* = 3 mice per experimental group. (**E**) Heatmap and enrichment of 26 DEGs unique to anti-CoV-2 EVs vs. uninfected groups. Data is representative of *n* = 3 mice per experimental group. (**F**) Volcano plots showing the differential gene expression analysis for the indicated treatment groups done for only 128 selected genes that were differentially expressed in SARS-CoV-2 only infected lungs experimental group (shown in panel B). The top 5 DEGs are highlighted. Data is representative of *n* = 3 mice per experimental group. (**G**) Heatmap of top DEGs identified in panel F, displaying their differential expression levels across the four experimental groups. Data is representative of *n* = 3 mice per experimental group. (**H**) Functional enrichment (GO/KEGG) of the top DEGs (*Lirf5*, *Irf2*, *Tnfrsf10b*, *Thbs1*, and *Sele*) shown in G. Data is representative of *n* = 3 mice per experimental group

We previously reported that unmodified, native NSC EVs reduced key inflammatory cytokines including, interleukin (IL)-1β and tumor necrosis factor (TNF)-α in poly I: C treated human macrophages [4]. To further explore these effects in vivo, RNA from infected lungs intranasally treated with anti-CoV-2 EVs were subjected to NanoString immune-transcriptomic profiling. Principal component analysis (PCA) of the gene expression profiles revealed distinct clustering in lungs from these experimental groups (Fig. 3A), with PC1 and PC2 showing 80.3% and 11.7% of the variance, respectively, demonstrating substantial differences between the experimental groups. This clustering pattern suggests that each experimental condition drives specific transcriptional programs, indicating unique underlying biological mechanisms. Differential gene expression analysis (DEG) identified genes that were regulated in each experimental condition. We found several up- and down-regulation genes in both the anti-CoV-2 EV and control EV treatment groups relative to uninfected and virus only control groups (Fig. 3B). Notably, 78 genes were differentially expressed across all EV treated and non-treated virally infected groups (Fig. 3C). The Circos plot provides a comprehensive overview of these 78 common DEGs and their functional annotations, indicating their involvement in various cellular compartments and biological processes, including membrane-associated and immune signalling pathways (Fig. 3D). This suggests that these DEGs may play a crucial role in mediating the response to SARS-CoV-2 infection. Importantly, we identified 26 DEGs unique only in the anti-CoV-2 EVs treated lungs group (Fig. 3E). These DEGs include downregulated genes that mediate the positive regulation of protein kinase activity, IL-1β and IL-8 production, as well as upregulated genes related to nucleoside biosynthetic process. These enriched pathways suggest a strong anti-inflammatory effect in virally infected lungs treated with anti-CoV-2 EVs. Furthermore, differential expression analysis (Fig. 3F) in anti-CoV-2 EVs and control EVs treated SARS-CoV-2 infected lungs of 128 virus genes identified as either up- or down-regulated in SARS-CoV-2 infected alone lungs (relative to uninfected control, Fig. 3B) identified several key genes, including *Lirf5*,* Irf2*,* Tnfrsf10b*, *Thbs1*, and *Sele*, which have been shown to be critical in modulating SARS-CoV-2 antiviral responses in the lungs [20]. Importantly, the differential expression levels of these key DEGs across the four experimental groups revealed similar expression levels between the uninfected and anti-CoV-2 EVs group (Fig. 3G), not between the virus only and control EV groups, suggesting that these key DEGs may contribute to the observed therapeutic effects of our anti-CoV-2 EVs. Finally, combining the functional enrichment analysis of these downregulated genes (Fig. 3H) with all our transcriptomic findings demonstrate that anti-CoV-2 EVs can modulate host cell immune responses, downregulate cytokine-related pathways, upregulate biosynthetic processes, and modulate cell death processes in the lungs of SARS-CoV-2 infected mice. qRT-PCR analysis confirmed that while SARS-CoV-2 infection dramatically increased pro-inflammatory cytokines in the lungs, treatment with anti-CoV-2 EVs significantly suppressed the expression of all measured markers, including IFN-α, IFN-β, IFN-γ, TNF-α, IL6, and MCP-1 (Figure S4). This anti-inflammatory effect was substantially more potent and consistent than that observed with control EVs, validating that our engineered EVs effectively mitigate the harmful pulmonary inflammation driven by the virus.

In this study, we provide evidence that the use of a viral targeting nanobody on the surface of EVs can directly neutralize viral infection both in vitro and in vivo. This is the first demonstration of the use of engineered EVs decorated with antiviral nanobodies for targeting SARS-CoV-2 infection in mice, let al.one using NSC derived EVs. Previous work using NSC derived EVs loaded with anti-SARS-CoV-2 asRNA showed efficacious antiviral effect in mouse lungs, albeit with an almost complete obliteration of viral infectious load [4]. Mechanistically, asRNA-mediated antiviral targeting relies on the transport of this RNA cargo into infected cells to be able to exert its antiviral silencing effect by directly binding to target viral mRNA molecules in the cytosol. Similarly, an intranasally delivered RNA interference (RNAi) platform using small-interfering RNAs (siRNAs) for targeting SARS-CoV-2 can achieve a similar antiviral potency in the lungs [21, 22]. This exposes the limitations associated with the use of an antiviral-nanobody EV approach when given therapeutically as it can only directly neutralize infectious viruses that are extracellular (i.e., during viral egress). However, it is important to highlight that the antiviral effect we observe with our anti-CoV-2 EVs was achieved with just two intranasal doses of EVs. We speculate that we can further maximise this lung antiviral effect if more daily doses are administered. We are cognizant of the limitations associated with intranasal EV delivery. Inhalable delivery of EVs by nebulisation may improve antiviral outcomes in the lower respiratory tract and perhaps improve mucociliary clearance in the lungs. Future work should test the effectiveness of nebulising anti-CoV-2 EVs in vivo.” Beyond direct antiviral targeting, the novelty in our work lies in the ability for NSC EVs to dampen SARS-CoV-2 driven lung inflammation. Our immune transcriptomic findings feature the added benefit that our targeting EVs offer as an anti-inflammatory agent. Indeed, an EV-based antiviral therapy that could also reduce tissue inflammation may be ideal for tackling severe COVID-19 situations where pulmonary ‘cytokine storm’, which leads to acute respiratory distress syndrome (ARDS) [23], is at its peak. This is especially important as anti-inflammatory agents can evade pulmonary tissue damage induced by the inflammatory response observed in patients with severe COVID-19. The clinical value of combination therapy with antiviral and anti-inflammatory drugs for the treatment of COVID-19 is well documented during the pandemic [1]. To date, we are unsure of the components that make NSC EVs possess inherent anti-inflammatory properties. A previous study that investigated the proteome and small microRNA (miRNA) content of NSC EVs speculated that its anti-inflammatory effects may be linked to certain miRNAs (miR-21-5p, miR-103a) and proteins (PTX3, hemopexin, Gal-3BP) [24]. Future work is needed to fully elucidate this. In saying this, we remain cautious as to not make strong claims to the observed anti-inflammatory activity of NSC EVs on SARS-CoV-2 infected lung tissues (Fig. 3). We merely observed immune-transcriptomic changes in lung infected tissue that remains to be verified at the protein and histological level. Our study has other limitations that need to be addressed in future studies. Though we have shown that HEK VHH72-EVs are equipotent across multiple SARS-CoV-2 variants in vitro [5], we need to demonstrate this in vivo. Our current study utilizes the ancestral SARS-CoV-2 infection model as we previously showed that infection with this variant resulted in a more severe disease in K18-hACE2 mice [22] and hence making appropriate for the experimental work we described here. Future work will also explore the impact of our anti-CoV-2 EVs on lung histopathology and function (e.g., respiration rate and oxygen saturation). Despite NSC EVs being relatively inert in intravenously [4] and intranasally (Fig. 2A) administered uninfected mice, a larger and more comprehensive pharmacological safety assessment, EV serum half-life determination and first-pass organ toxicity is warranted in future investigations.

In conclusion, our EV-based strategy paves way for a new antiviral modality that can be applied to target not only SARS-CoV-2 but also other viral diseases. EVs are also the ideal ‘natural’ vehicle for delivering RNA therapeutics as demonstrated recently for SARS-CoV-2 targeting [4], and provide a unique alternative to lipid nanoparticles (LNPs). EVs are modular and can be equipped and ladened with multiple arsenals, encapsulated or on its external surface, to neutralize pathogens, target specific tissues or enhance endosomal escape and unloading of therapeutic cargo into target cells. Importantly, intranasal delivery of EVs further minimizes the toxicity concern in non-target organs and allows EVs to localize to the upper and lower respiratory tracts for a short period of time (EVs are no longer present in lungs by 24 h) (Fig. 2A), which is ideal for targeting an acute viral infection (i.e., SARS-CoV-2), bypassing the concern for potential long-term modified EV (e.g., VHH-ladened EVs) exposure-related tissue adverse reactivity. Moreover, studies in human subjects from numerous phase I clinical trials further underscores the safe and non-invasive utility of intranasally delivered EVs for various pulmonary conditions [25]. Nanobody-loaded EVs can be scaled up for clinical or commercial applications, but it requires addressing challenges related to production, purity, and cost-effectiveness. However, it is important to note that scaling up their production and purification remains a key hurdle [26]. Though molecular engineering cells, as we have done, to produce EVs ladened with targeting moieties may offer a more scalable alternative, achieving EVs at high purity remains challenging in the manufacturing industry. The cost-effective ultracentrifugation remains time-consuming and has the propensity to be impure. On the other hand, size-exclusion chromatography methods may achieve higher purity but are associated with high costs (e.g., labor-intensive). Moreover, batch variability and scalability issues remain unresolved. However, the EV purification and isolation techniques are improving over time as companies are continually developing and advancing these technologies to meet the emerging and rapid interest in EV therapeutics. Despite this, our work demonstrates that molecularly engineered EVs could potentially become a new platform for delivering antiviral therapeutics.

## Electronic supplementary material

Below is the link to the electronic supplementary material.

Supplementary Material 1

## Data Availability

All data generated or analyzed during this study are included in this manuscript.
