# Supplementary material for: Intranasal delivery of engineered anti-SARS-CoV-2 extracellular vesicles therapeutically represses lung infection and inflammation
Source: Drug Deliv Transl Res. 2025 Jul 17;15(11):4115–25. doi: 10.1007/s13346-025-01922-9 (PMC12507999; doi:10.1007/s13346-025-01922-9)
Supplement: Supplementary file 1 — Supplementary Material 1 [file 13346_2025_1922_MOESM1_ESM.docx]

**Supplementary figures and figure legends**


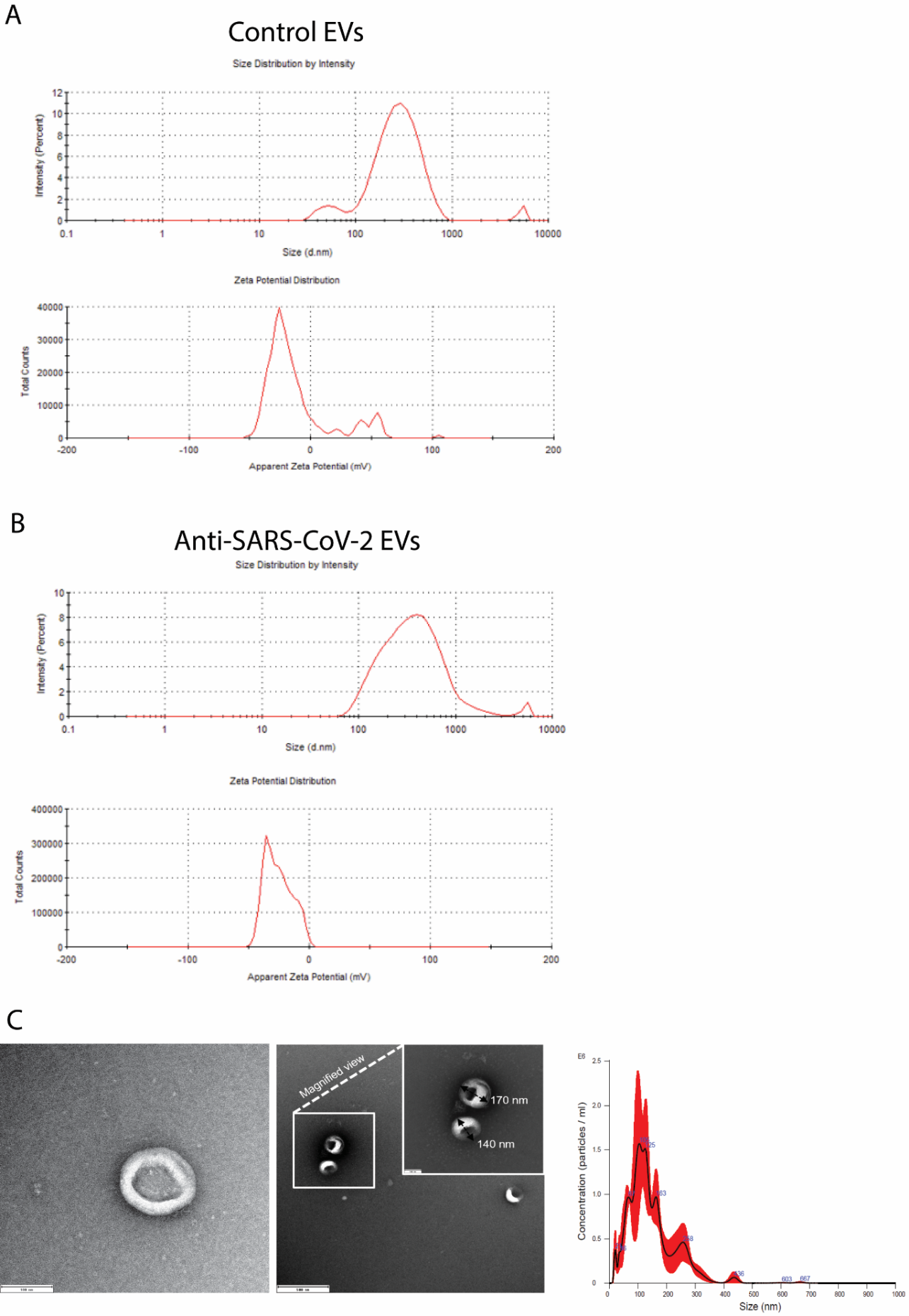


**Figure S1** The particle size, polydispersity index (PDI) and zeta potential of EVs were obtained using Zetasizer Nano ZS following appropriate dilution in PBS. All measurements were carried out at room temperature. Particle peak intensities by size for LNP formulations were measured using dynamic light scattering (DLS) curves for (A) control EVs and (B) anti-SARS-CoV-2 EVs. (C) EVs were also subjected to TEM and NTA analysis.


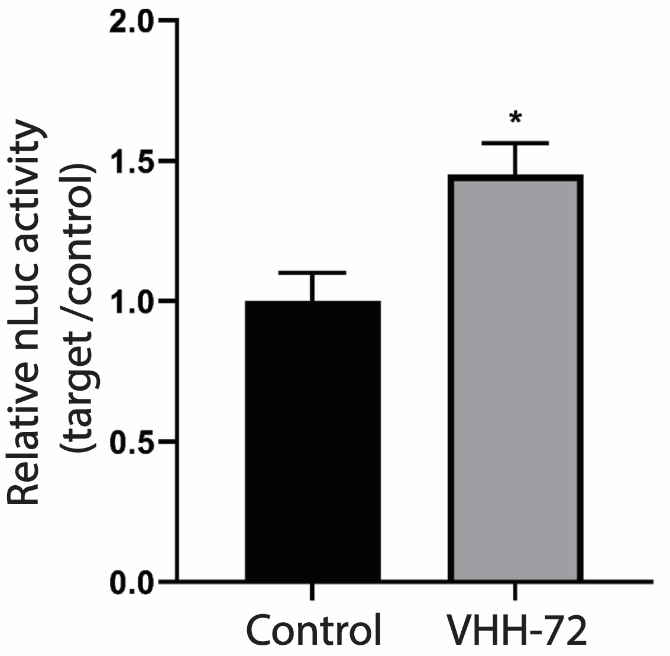


**Figure S2** HEK293 cells were transfected with a SARS-CoV-2 spike protein-expressing vector and treated with VHH72-CD63 EVs (anti-CoV-2 EVs). The levels of Nluc were assessed at 4 h post-exposure and normalized to untransfected HEK293 cells and made relative to the CD63 control (Control EVs). Error bars represent SD (n=3), ∗p < 0.05 Student’s t test compared to control.


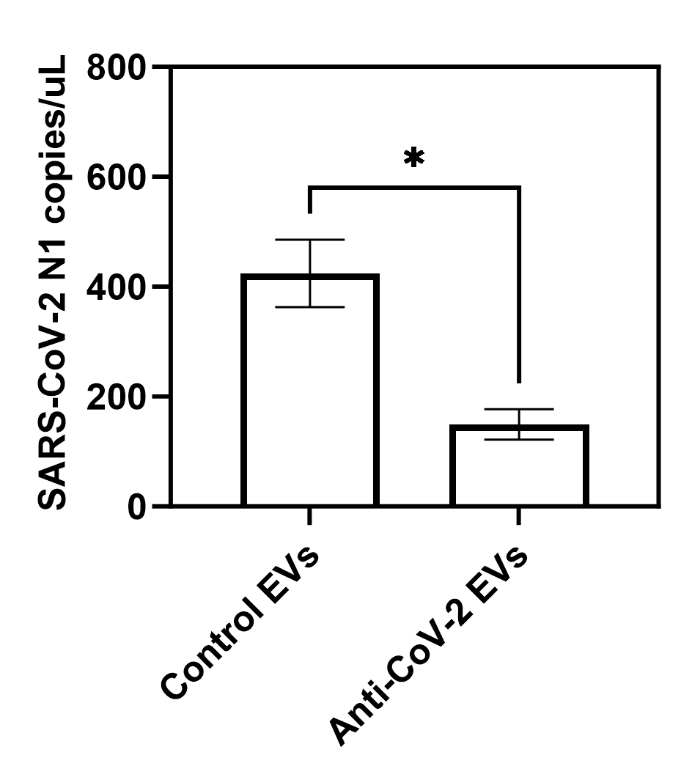


**Figure S3** Lung tissues were subjected to RNA extraction and viral copy numbers determined by digital droplet PCR against SARS-CoV-2 N gene. Bars represent the mean viral copy numbers/ µl and error bars represent the SEM (4 mice). *p<0.05, One-way ANOVA test.


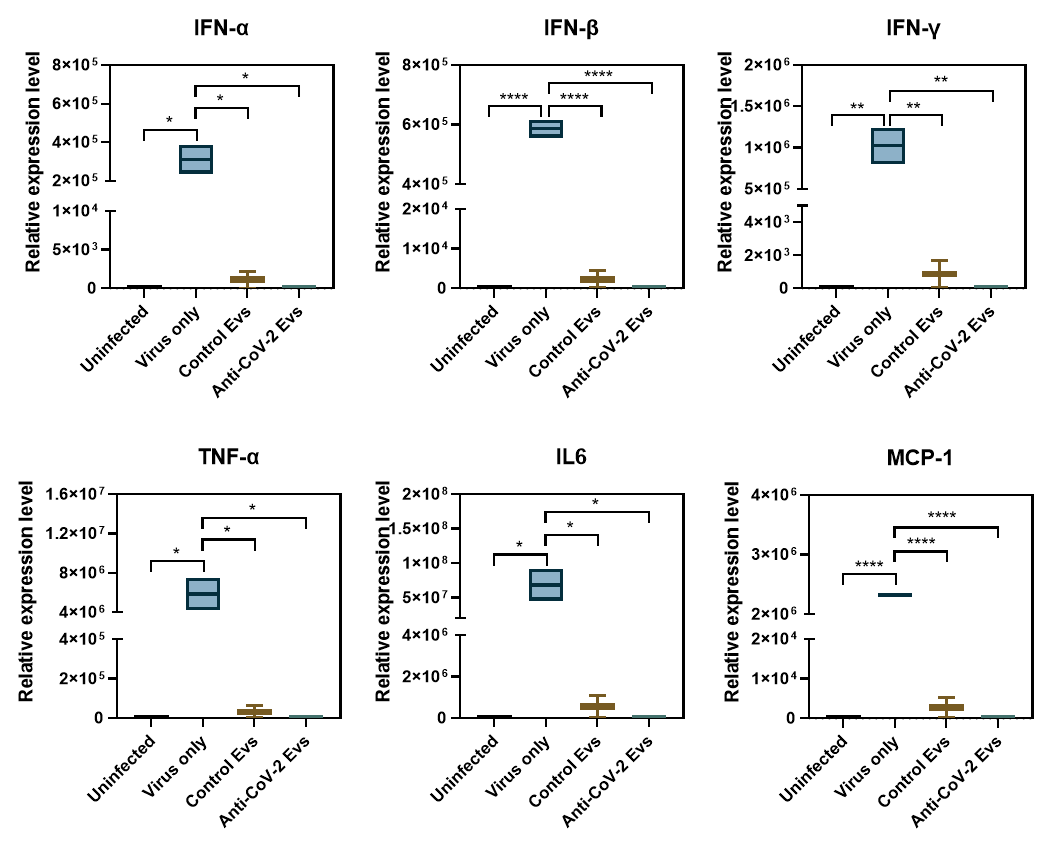


**Figure S4** Analysis of inflammatory cytokine levels in the lungs of SARS-CoV-2-infected mice. The mRNA levels of IFN-α, IFN-β, IFN-γ, TNF-α, IL-6, and MCP-1 genes were determined by qRT-PCR. Relative expression levels were normalized to β-actin levels. Values are the mean ± SEM (n = 3 mice per group). *, p < 0.05; **, p < 0.001; ****, p < 0.0001.
